# The Organization of Choroidal Arteries in Normal Eyes

**DOI:** 10.1016/j.xops.2026.101294

**Published:** 2026-06-18

**Authors:** Achille Barre, Côme LeGall, Céline Chaumette, Daniela Castro Farias, Sarah Mrejen, Leo Puyo, Michael Atlan, Michel Paques

**Affiliations:** 1 Paris Eye Imaging Group, Clinical Investigation Center 1423, Quinze-Vingts Hospital, INSERM-DHOS, and Sorbonne Université, Paris, France; 2 Institut Langevin, Paris Sciences & Lettres University, École Supérieure de Physique et de Chimie Industrielles, Paris, France

**Keywords:** Choroid, Laser Doppler holography, Long posterior ciliary artery, Paraoptic arteries, Submacular artery

## Abstract

**Objective:**

To determine the organization of normal human choroidal arteries using a combination of laser Doppler holography (LDH) and OCT.

**Design:**

Cross-sectional clinical study.

**Subjects:**

One hundred thirty-two eyes of 74 healthy subjects.

**Methods:**

By combining images from a prototypic LDH system and from OCT, we documented the 3-dimensional disposition of choroidal arteries from their emergence from ciliary arteries to precapillary arterioles.

**Main Outcome Measures:**

Distribution, diameters, and pathways of choroidal arteries.

**Results:**

A notable intereye variability in the disposition of arteries was found, in which dominant patterns were identified. A short ciliary artery emerging within 1000 μm from the fovea was identified in 63% of eyes, giving rise to a horizontal submacular artery (SMA; mean diameter 119.7 ± 24.9 μm) oriented temporally. The detection rate of an SMA was inversely correlated with choroidal thickness, that is, SMAs were more often identified in thinner choroids. Among cases with a detectable SMA, its diameter positively correlated with choroidal thickness (*P* < 0.003). Long posterior ciliary arteries were identified in 43% of eyes and emerged at a mean distance of 4820 ± 1567 μm temporal to the fovea (mean diameter 115 ± 41 μm). Paraoptic arteries radiated from the margins of the optic nerve head, some of which perfused the macula. Disseminated axially oriented arterioles perfusing precapillary arterioles were detected from first-order arteries.

**Conclusions:**

Using a combination of LDH and OCT in healthy eyes, we identified recurrent organizational patterns of choroidal arteries in normal eyes, including SMAs, long posterior ciliary arteries, paraoptic arteries, and precapillary complexes. Characterizing the physiologic variants of the disposition of choroidal arteries helps to provide a reference framework that will enable the identification of disease-related changes.

**Financial Disclosure(s):**

Proprietary or commercial disclosure may be found in the Footnotes and Disclosures at the end of this article.

Choroidal vessels are the major source of oxygen and metabolites for photoreceptors.^1^^,^^2^ Understanding the vascular organization of the choroid is crucial to elucidate its physiopathology. However, the complex, 3-dimensional vascular mesh of the vasculature, which is a major determinant of choroidal volume,^3^ defies conventional approaches. Characterization of the choroidal microvasculature by histology has inherent limitations, notably the difficulty in capturing the 3-dimensional disposition owing to postmortem flattening of vessels. Choroidal arteries are downstream of short posterior ciliary arteries (SPCAs) and long posterior ciliary arteries (LPCAs), themselves derived from the ophthalmic artery. Short posterior ciliary arteries penetrate the sclera in the posterior pole and in the perioptic area; arteries from perioptic SPCA perfuse the Zinn–Haller circle. Emerging from the Zinn–Haller circle, histology has reported the presence of arteries perfusing the choroid.^4^ Histology has shown that choroidal vessels exhibit an anteroposterior gradient in size, conventionally separated into 3 compartments: the choriocapillaris, the Sattler layer containing small and medium vessels, and the Haller layer comprising large vessels.^5^ This classification has been challenged because of the absence of a clear anatomic demarcation between the Haller and Sattler layers.^6^ On fundus photographs, choroidal vessels are rarely seen, and even indocyanine green angiography, which is very efficient at characterizing midsized and large veins, cannot reliably sort out the arterial arborization.

The advent of OCT increased the capability to reliably, quantitatively, and noninvasively document the choroidal vasculature. This revealed that the thickness of the choroid exhibits significant interindividual variability, which correlates with axial length and aging.^7^ OCT imaging led to the identification of so-called pachychoroid diseases, a clinical entity associated with enlarged choroidal vessels.^8^ Yet, a comprehensive understanding of the organization of choroidal circulation remains elusive; indeed, fundamental features of the organization of choroidal vasculature, such as the differentiation of arteries from veins and their spatial arrangement, remain largely incomplete.

Recently, we used a new technology, laser Doppler holography^9^^,^^10^ (LDH), that enables the identification of human choroidal vessels in vivo noninvasively. Because the outputs of LDH from a single data set are customizable, during postprocessing, it is possible to threshold the Doppler shift at different values to differentiate high flow (i.e., arteries) from low flow (i.e., small arterioles and veins). Combining LDH and OCT allowed us to demonstrate that first- and second-order choroidal arteries are located along the sclerochoroidal interface and are generally posterior to veins^11^ and that in most healthy eyes, a horizontal submacular artery (SMA) is present. Here, to further explore the choroidal angioarchitecture, we performed a systematic analysis of choroidal arteries from their emergence from the sclera to precapillary arterioles.

## Methods

Clinical procedures adhered to the tenets of the Declaration of Helsinki and were approved by an institutional review board (ethics committee, Comité de Protection des Personnes Sud Mediterrannee IV [Montpellier, France]). The study was registered at ClinicalTrials.gov (NCT04129021). The imaging charts of 74 patients were reviewed. All subjects received both oral and written information and signed a consent form before inclusion. Among the 74 subjects, the images from 16 eyes were excluded because of image quality and/or unilateral disease.

The LDH prototype has been previously described.^9^^,^^10^ Briefly, it consists of an optical interferometer using flood illumination from a 785 nm laser diode. The interferograms are recorded using a high-speed camera (frame rate: 39 kHz) at 512 × 512 pixel resolution over a 40° field of view. From each data set, Doppler signal maps are generated by numerically propagating the diffracted speckle pattern to the choroidal plane. Adjusting the Doppler shift (typically 35–39 kHz for high velocities and 1–10 kHz for low velocities) enables the capture of different flow velocities. Stepwise changes in the Doppler shift allowed for tracking the branching of arteries down to the precapillary arterioles. Retinal and choroidal arteries were both in focus in the same image in most eyes; therefore, the same focus setting was used to track vessels at different depths in the choroid. The postprocessing of images was customized to optimize the detection of each type of vessel on a case-by-case basis. High-frequency maps were first used to identify large vessels; lower-frequency maps were used to follow the vascular continuity into smaller branches. Measuring the asymmetry of the Doppler power signal (so-called M1 imaging^12^) allowed us to distinguish anterograde from retrograde Doppler shifts and thereby identify axially oriented vessels. Image montaging and registration were performed using i2k software (DualAlign, LLC).

Transverse and en face OCT scans were performed using commercial systems, the PlexElite 9000 (Zeiss) or the Spectralis SLO-OCT (Heidelberg Engineering). Raster scans centered on the macula were acquired and viewed in transverse and en face modes. On B-scans, first-order arteries were identified based on their location along the sclerochoroidal interface, their connection to ciliary arteries, and the presence of a periarterial avascular space (termed the *halo*).^11^ The distance from the fovea to the emergence of the SMAs and LPCAs, as well as their diameter and the subfoveal choroidal thickness, were measured using built-in calipers of the Spectralis system. The diameter of the SMA was measured immediately after its emergence from the SPCA, that is, when the SMA made a 90° turn and was located along the plane of the sclerochoroidal interface. Measurements were performed in a masked fashion by 2 authors (A.B. and M.P.); discrepancies were resolved by a third author (S.M.).

## Results

**Figure 1:**
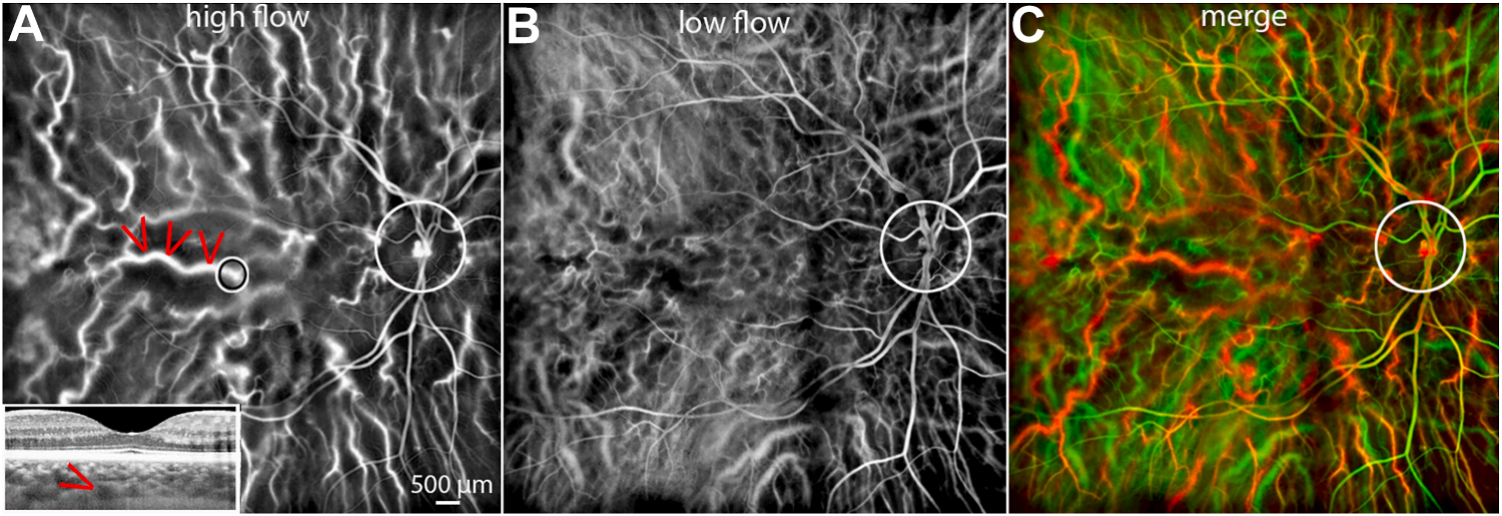
Representative large field laser Doppler holography imaging of a 33-year-old man (refraction –3 diopter; choroidal thickness 240 μm). The field of view is 40**°**. **A, B,** High- and low-velocity maps showing, respectively, arteries (Doppler shift 26–39 kHz) and small arterioles and veins (2–10 kHz). The small circle indicates the fovea, the large circle the optic nerve, and the red arrowheads the submacular artery (SMA). The insert in A is a vertical OCT scan showing the SMA surrounded by an avascular halo. In **C**, the red-green merged image demonstrates minimal overlap between arteries and veins.

**Figure 2:**
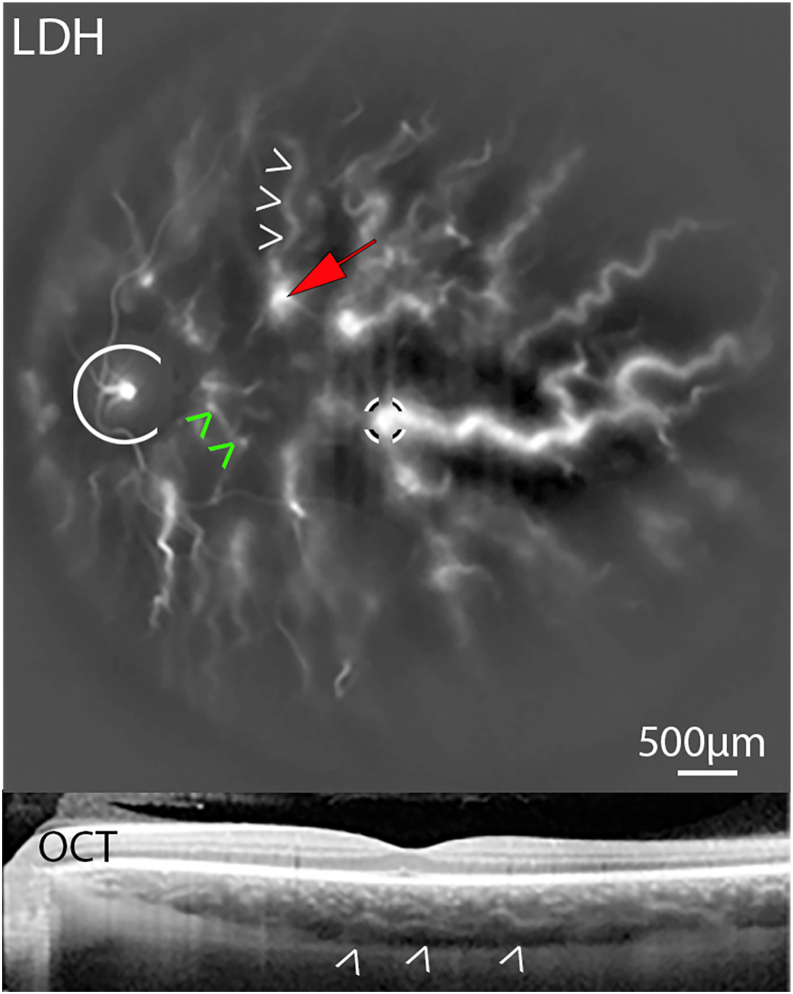
Laser Doppler holography imaging (Doppler shift 35–39kHz) of a 28-year-old man with emmetropia (central choroidal thickness 465 μm). The field of view is 40°. The fovea is in the central circle. The red arrow indicates a short ciliary artery from which emerges a perimacular artery (white arrowheads); green arrowheads show a paraoptic artery. The OCT confirms the location of the submacular artery along the sclerochoroidal interface (arrowheads).

**Figure 3:**
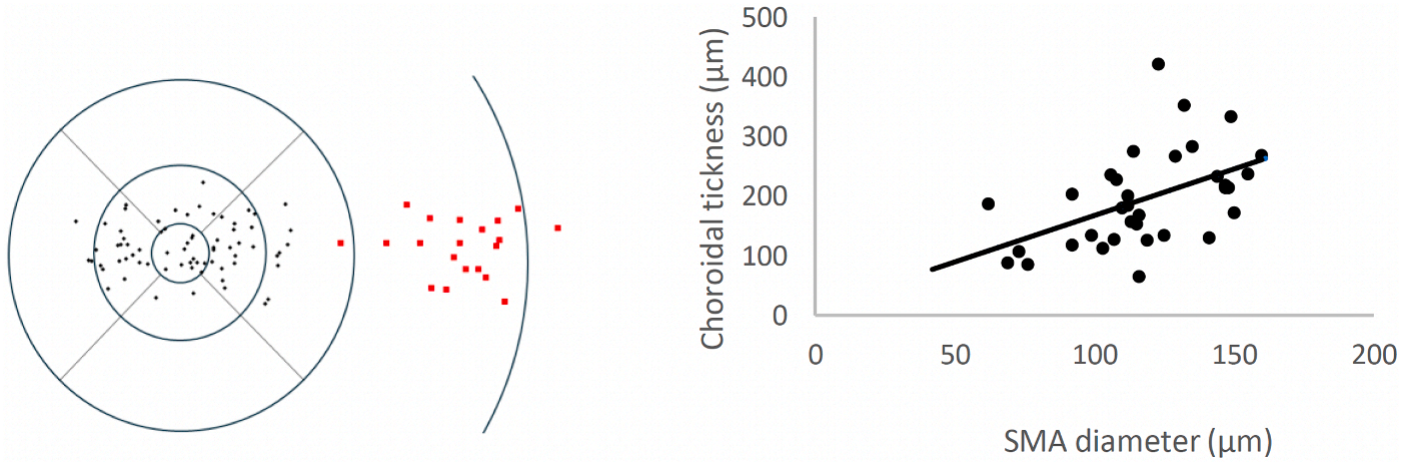
**Left,** distribution of the emergence of submacular arteries (SMAs; black dots) and long posterior ciliary arteries (red dots). The ETDRS grid displays 0.5-, 1.5-, and 3-mm radii circles centered on the fovea; the temporal arc is 6 mm from the fovea. All data are presented as left-eye equivalents. **Right,** correlation between the diameter of the SMA and central choroidal thickness (*r* = 0.4; *P* < 0.003).

The characteristics of the included population are summarized in Table 1. Figures 1 and 2 show representative LDH images of choroidal vessels. Adjusting Doppler shift thresholds allowed for efficient discrimination between arteries and veins, as shown by the limited overlap on merged images. A short ciliary artery emerging within 1000 μm from the fovea, giving rise in most eyes to a horizontal SMA oriented temporally, was identified in 63% of eyes. In most eyes in which an SMA was detected, the latter was the largest choroidal artery in the posterior pole (example in Fig 2). The mean ± standard deviation diameter of the SMAs was 119.7 ± 24.9 μm; there was a positive correlation between choroidal thickness and the diameter of the SMA (*r* = 0.4; *P* < 0.003; Fig 3). However, a limit to this correlation is that the SMA was less visible in thicker choroids. In eyes with a detectable SMA, the mean choroidal thickness was 196.1 ± 79.8 μm, compared with 263.4 ± 109.3 μm in eyes without detectable SMA (*P* = 0.001). This difference may cause a bias reinforcing the correlation because it can be assumed that small SMAs in thick choroids are more likely to be undetected.

**Table 1:** Clinical Characteristics of the Included Population (*N* = 74)

Clinical Characteristic	Value
Age (yrs ± SD) (range)	54.9 ± 19.8 (21–86)
Sex (female), *n* (%)	34 (45.9%)
Refractive error (spherical equivalent ± SD) (range)	–0.49 ± 2.62 (–10.25 to +5.00)
Axial length (mm) ± SD (range)	24.0 ± 1.5 (21.81–29.42)
Subfoveal choroidal thickness (μm ± SD) (range)	248.8 ± 102.8 (50–539)

SD = standard deviation.

**Figure 4:**
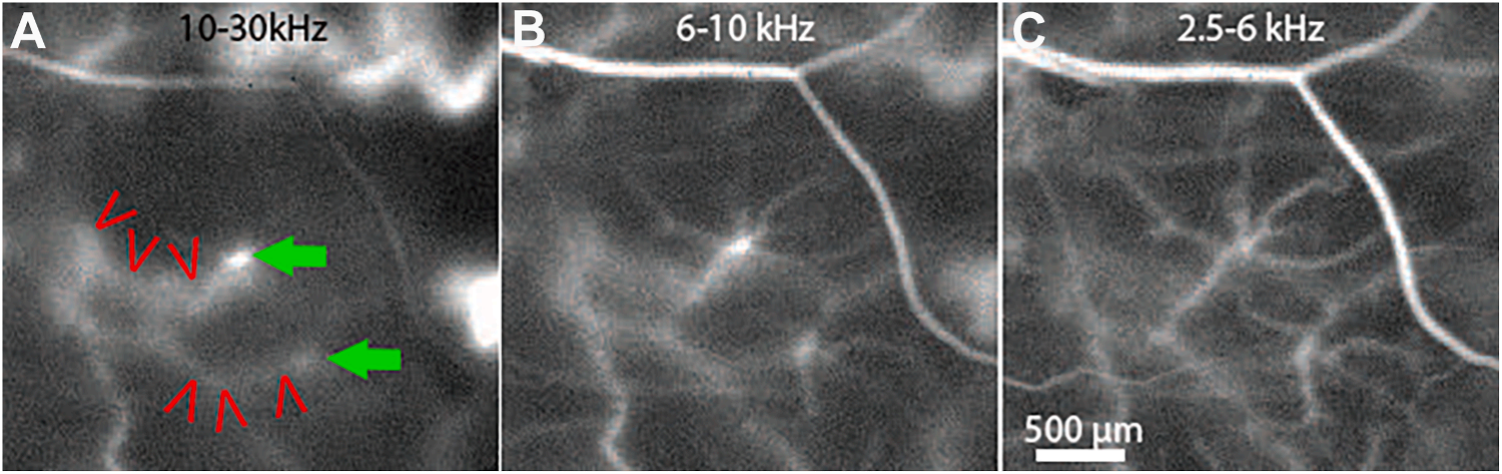
Characterization of precapillary complexes by stepwise Doppler shift thresholding. **A,** Laser Doppler holography imaging of second-order arterioles (red arrowheads) temporal to the macula, giving rise to 2 axial arteries (green arrows). **B** and **C** show 2 adjacent precapillary complexes.

**Figure 5:**
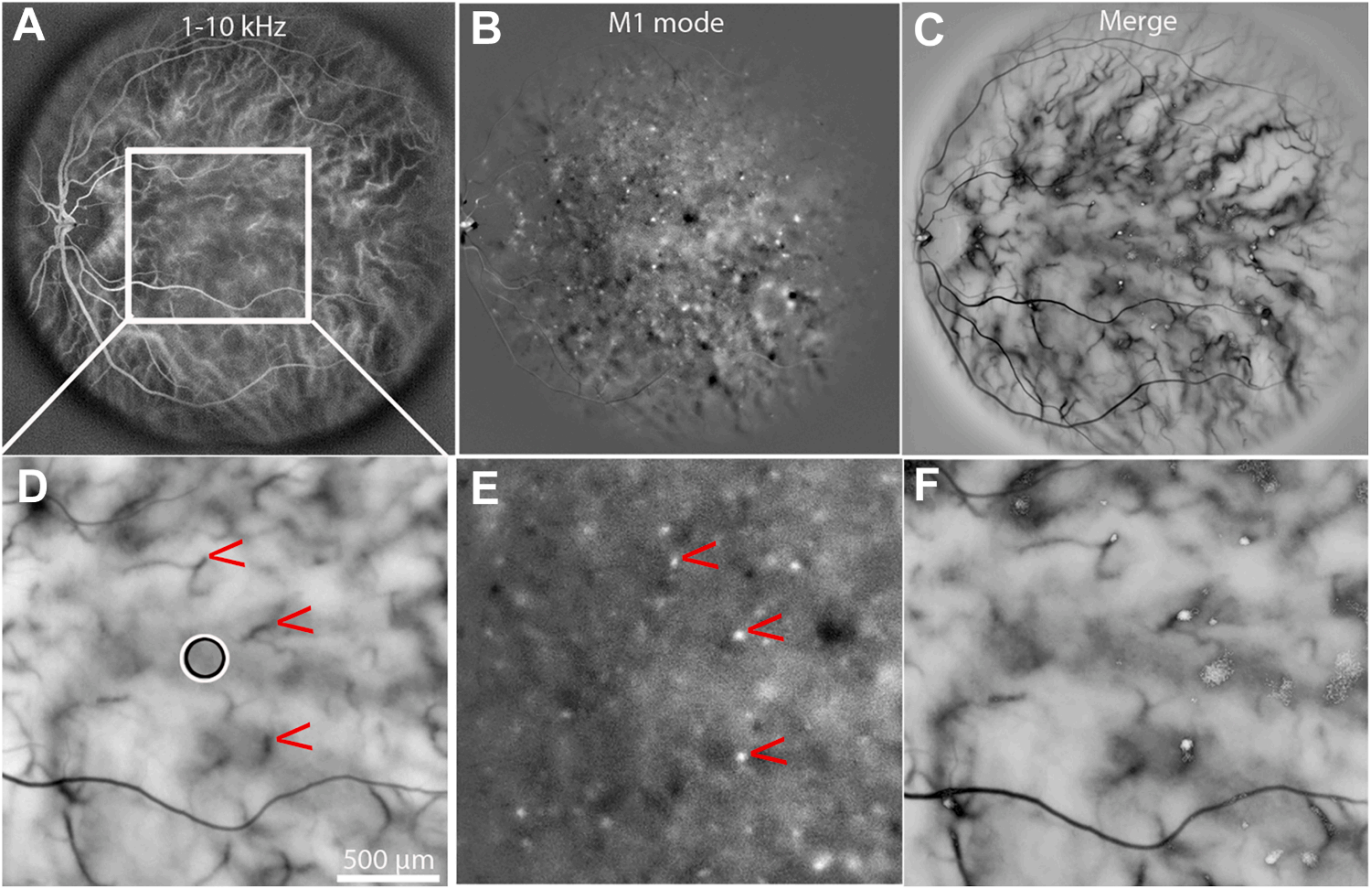
Organization of precapillary arterioles (same eye as in Figure 2). Panels **A–C** are magnified in **D–F,** respectively. The field of view in panels **A–C** is 40°. **A, D,** Laser Doppler holography image showing precapillary arterioles (examples are indicated by red arrowheads in **D**, which have been inverted to improve clarity; the circle indicates the fovea). **B,** M1 imaging showing anterograde axial flow. **C,** Merged images of A and B demonstrating that axial arterioles (in white) perfuse precapillary arterioles.

The continuity between second-order arterioles, axial arterioles, and precapillary arterioles was documented by stepwise variations of Doppler shift thresholds (Fig 4). This allowed documentation of the connections between first-order arteries and precapillary arterioles. The smallest detectable arterioles formed bouquets of 2 to 6 precapillary arterioles, with each of these bouquets covering a surface area of 0.5 to 1 disc diameter. We termed the association of an axial arteriole and the bouquet of precapillary arterioles a precapillary complex. Figure 5 shows the distribution of precapillary complexes in the posterior pole. Axial flow maps (M1 imaging) facilitated the identification of axial arterioles perfusing precapillary complexes in the macula (Fig 5).

**Figure 6:**
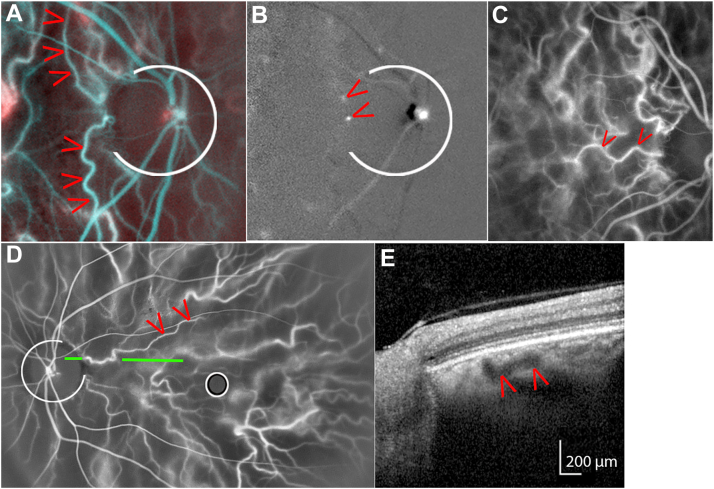
**A,** Example of 2 vertical paraoptic arteries. **B,** Axial flow map (M1 imaging) showing their origin. In the optic disc, note the opposite signal of the central artery (white) and vein (black), demonstrating directional discrimination of inflow versus outflow. **C, D,** Examples of paraoptic arteries reaching the macular area (circle indicates the fovea). **E**, The OCT scan along the green line in D, showing the paraoptic artery.

**Figure 7:**
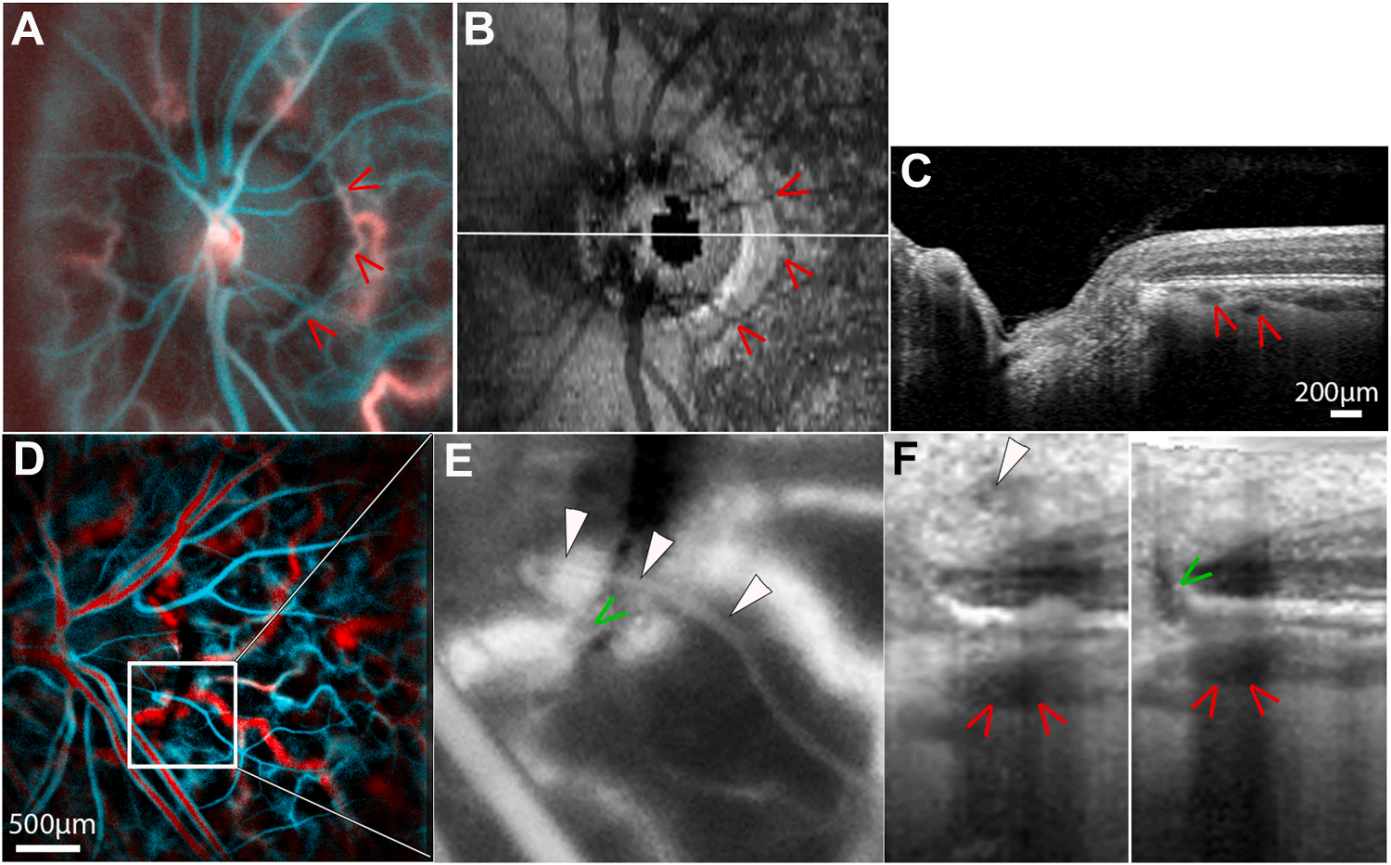
**A–C,** Paraoptic arteries circling the optic nerve head. **A,** Laser Doppler holography (LDH) image of a circular paraoptic artery, possibly the Zinn–Haller circle; **B,** en face OCT; **C,** transverse OCT along the line in (B) showing the subretinal pigment epithelium location of paraoptic arteries. **D–F,** Emergence of a cilioretinal artery. **D,** The LDH image; **E,** magnification showing the cilioretinal artery (white arrowhead). The green arrowhead shows the connection between the cilioretinal artery and the paraoptic artery on the LDH image and on the transverse OCT (F).

From the margins of the optic disc emerged 4 to 8 radial arteries (Fig 6), the diameters of which ranged from 60 to 82 μm (± 24.2 μm). They ran over distances from a few 100 μm to several millimeters. We propose to term these arteries *paraoptic arteries*. Those emerging temporal to the disc could reach the macular area (example in Fig 6C, D). In 1 eye, a paraoptic artery circling the optic nerve head was detected both by LDH and en face OCT (Fig 7A–C). This most likely corresponded to the Zinn–Haller circle. Cilioretinal arteries, emerging from the edge of the optic disc and perfusing a variable surface area of the retina, are commonly found in normal eyes. Among the 3 cases of cilioretinal arteries examined by LDH, their emergence colocalized with those of paraoptic arteries (Figs 7D–F), suggesting that cilioretinal and paraoptic arteries may be branches from the same short ciliary arteries and/or that cilioretinal arteries themselves originate from a paraoptic artery.

**Figure 8:**
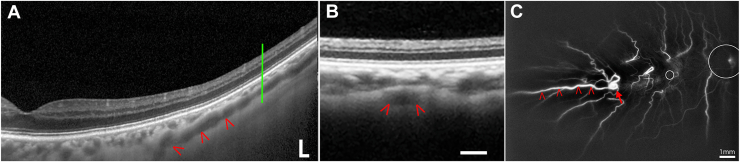
The OCT imaging of a long posterior ciliary artery (LPCA; arrowheads). **A,** Horizontal scan; **B,** vertical scan along the green line in A; note the periarterial halo (bars in A and B, 200 μm). **C,** Laser Doppler holography imaging of an LPCA (arrowheads). Two images were merged to achieve a 60° field of view. Note the focal arterial tortuosity (red arrow) at its emergence. Circles indicate the optic nerve and fovea (bar, 1 mm). See also Figure S1.

An LPCA (Fig 8, Fig S1, available at www.ophthalmologyscience.org) was identified in 43% of eyes. OCT had a better sensitivity to detect LPCAs than LDH. In eyes in which LPCAs were detected, they emerged at a mean ± standard deviation distance of 4,820 ± 1,567 μm temporal to the fovea. The LPCAs followed a less tortuous path than the SMAs. We found no evidence of branching of LPCAs. Similar to first-order ciliary arteries, LPCAs were located along the sclerochoroidal interface, embedded in an avascular space. The mean diameter of the LPCAs was 115 ± 41 μm. The site of emergence of the LPCAs often colocalized with focally tortuous arterioles, which seemed to originate from the LPCA itself (Fig 8).

## Discussion

Thanks to the unique capability of LDH to distinguish arteries from veins, we provide here a systematic in vivo characterization of the dominant patterns of choroidal arterial circulation. We confirm here that a horizontal first-order artery with a first meander oriented upward, emerging and/or running close to the fovea, which we termed an SMA, can be identified in most eyes. Hence, the presence of an SMA is one of the most common features of normal eyes. In the present report, we further explored the arterial arborescence of the choroid, which allowed us to propose refinements of the choroidal angioarchitecture, thanks to the identification of novel or rarely clinically documented features such as paraoptic arteries, LPCAs, and the organization of precapillary arterioles.

Exploring the physiologic variability and its correspondence with choroidal thickness is a first, necessary step before the characterization of pathologic choroidal vasculature. Histology cannot capture the spectrum of physiologic variations of the organization of choroidal vessels. Identifying anatomic models of complex structures requires identifying dominant organizational patterns; in the case of choroidal vessels, it is a challenging task because there is notable interindividual variability. Although there were dominant patterns for each type of choroidal artery, such patterns were infrequently simultaneously present in a given eye.

Our finding of a correlation between the diameter of the SMA and choroidal thickness suggests that the latter may be positively correlated with choroidal blood flow; in other words, thicker choroids may have higher blood flow. An important limit to our conclusions regarding this is that subjects in whom an SMA was not identified had, on average, a thicker choroid than those in whom an SMA was found. The apparent paradox of a lack of detection of large arteries in thicker choroids may be due to light dispersion because of their position deep in the choroid, and, paradoxically, to a higher flow velocity, which may be out of reach of the camera.

Doppler imaging is highly sensitive to detecting axial displacement of erythrocytes. Selecting positive or negative Doppler shifts from the power spectrum density histogram enables the extraction of maps of axial flow. We applied this to the analysis of the choroidal circulation, enabling the visualization of axial arteries and providing access to the 3-dimensional arrangement of choroidal vessels. Indeed, because first- and second-order arteries reside in the Haller layer along the sclerochoroidal interface, axial flow maps identify arteries branching from deep vessels and traversing the Sattler layer to reach the choriocapillaris. By combining low-velocity maps and axial flow maps, we documented the connections between first- and second-order arteries and precapillary arterioles. This revealed a previously unreported feature, namely, a central axial arteriole from which radiate the precapillary arterioles. We postulate that these radiating arterioles perfuse several lobules of the choriocapillaris.^5^^,^^13^, ^14^, ^15^ These bouquets of diverging arterioles form a supralobular organization, paving the posterior pole with bouquets of precapillary arterioles covering an area approximately 1 disc diameter wide, for which we propose the term *precapillary complexes*.

Paraoptic arteries have been described histologically as clusters of short posterior ciliary arteries perfusing the Zinn–Haller circle from which secondary branches penetrate the choroid.^4^^,^^16^^,^^17^ To our knowledge, there has been no previous report of in vivo imaging of these vessels. We found that paraoptic arteries have a generally radial pattern, extending over several millimeters in all directions, comprising the posterior pole. Indeed, some were directed toward the macula, hence potentially contributing to the perfusion of submacular choriocapillaries. Cilioretinal arteries emerged from the margins of the optic nerve head jointly with 1 or 2 paraoptic arteries, suggesting a common origin. Imaging of paraoptic arteries may also offer the possibility of exploring the Zinn–Haller circle, as suggested in one of our cases. This would be of the highest interest for a better understanding of optic nerve ischemia.^18^

We also characterized the disposition of LPCAs. A unique, large artery running horizontally toward the periphery was unequivocally identified in less than half of the examined eyes. We also found that OCT outperformed LDH for the detection of LPCAs. A possible explanation for this is that visualization of the LPCAs may be impaired by the periarterial halo, which may dim the LDH signal. Although the diameter of LPCAs was comparable to that of SMAs, their path was less tortuous. They were apparently devoid of branching; it is therefore unclear whether LPCAs contribute to the perfusion of the choriocapillaris. At their emergence, LPCAs often showed focal arterial tortuosity, a novel finding whose significance is uncertain.

**Figure 9:**
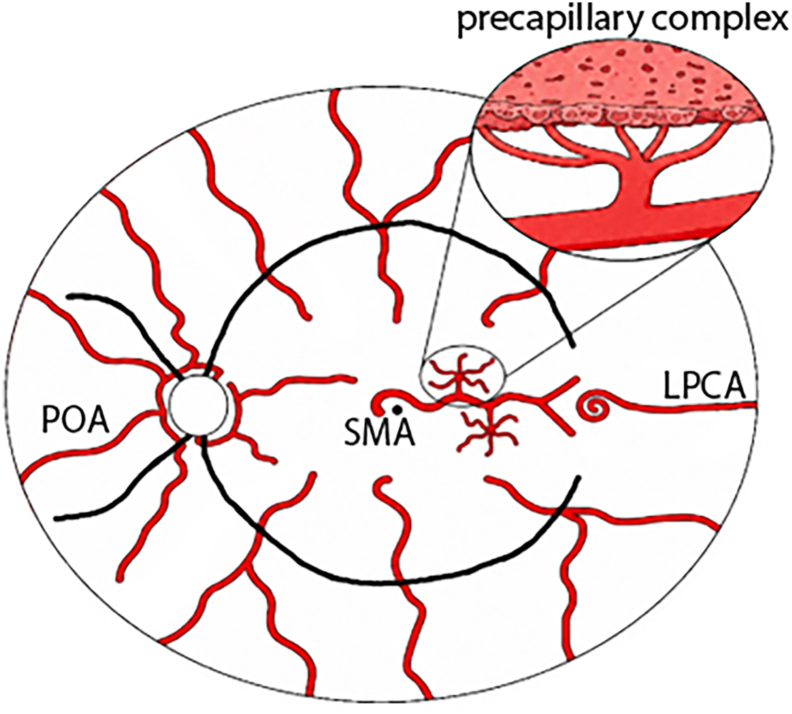
Proposed schematic of the most common disposition of choroidal arteries. LPCA = long posterior ciliary artery; POA = perioptic artery; SMA = submacular artery.

Keeping in mind the technical limitations of the LDH and the interindividual variability, these results led us to propose the following model (Fig 9): In the horizontal raphe are an SMA and an LPCA, both running temporally. The origin of the LPCA often shows focal tortuosity. Around the macula, multiple perimacular arteries radiate in all directions toward the periphery. Paraoptic arteries radiate from the margins of the optic nerve head. First-order arteries are located along the sclerochoroidal interface, generally posterior to veins; from first- and second-order arteries, transverse (axial) arterioles traverse the choroid to perfuse precapillary complexes that pave the posterior pole and supply choriocapillaris lobules.

The limits of our findings are related to the prototypic nature of LDH technology, which currently lacks a standardized acquisition procedure. The effect of the field of view, of the anatomic characteristics of the eyes, and of the focus plane of image reconstruction are poorly understood. There are also important issues related to the difficulty of exploring eyes with a pachychoroid because of signal attenuation. Improvements in the hardware and in postprocessing procedures are expected. Future studies should address expanding these findings, in particular, the relationship with choroidal thickness and the characterization of the different types of arteries.

In conclusion, we identified recurrent organizational patterns of choroidal arteries in normal eyes that may help to better understand choroidal vascular diseases. Further explorations are needed to grasp the spectrum of choroidal angioarchitecture in the thinnest and thickest choroids and to complement this with the determination of the spatial disposition of veins.
