# Supplementary material for: The Organization of Choroidal Arteries in Normal Eyes
Source: Ophthalmol Sci. 2026 Jun 18;6(9):101294. doi: 10.1016/j.xops.2026.101294 (PMC13487285; doi:10.1016/j.xops.2026.101294)
Supplement: Supplementary file 1 [file mmc1.pdf]

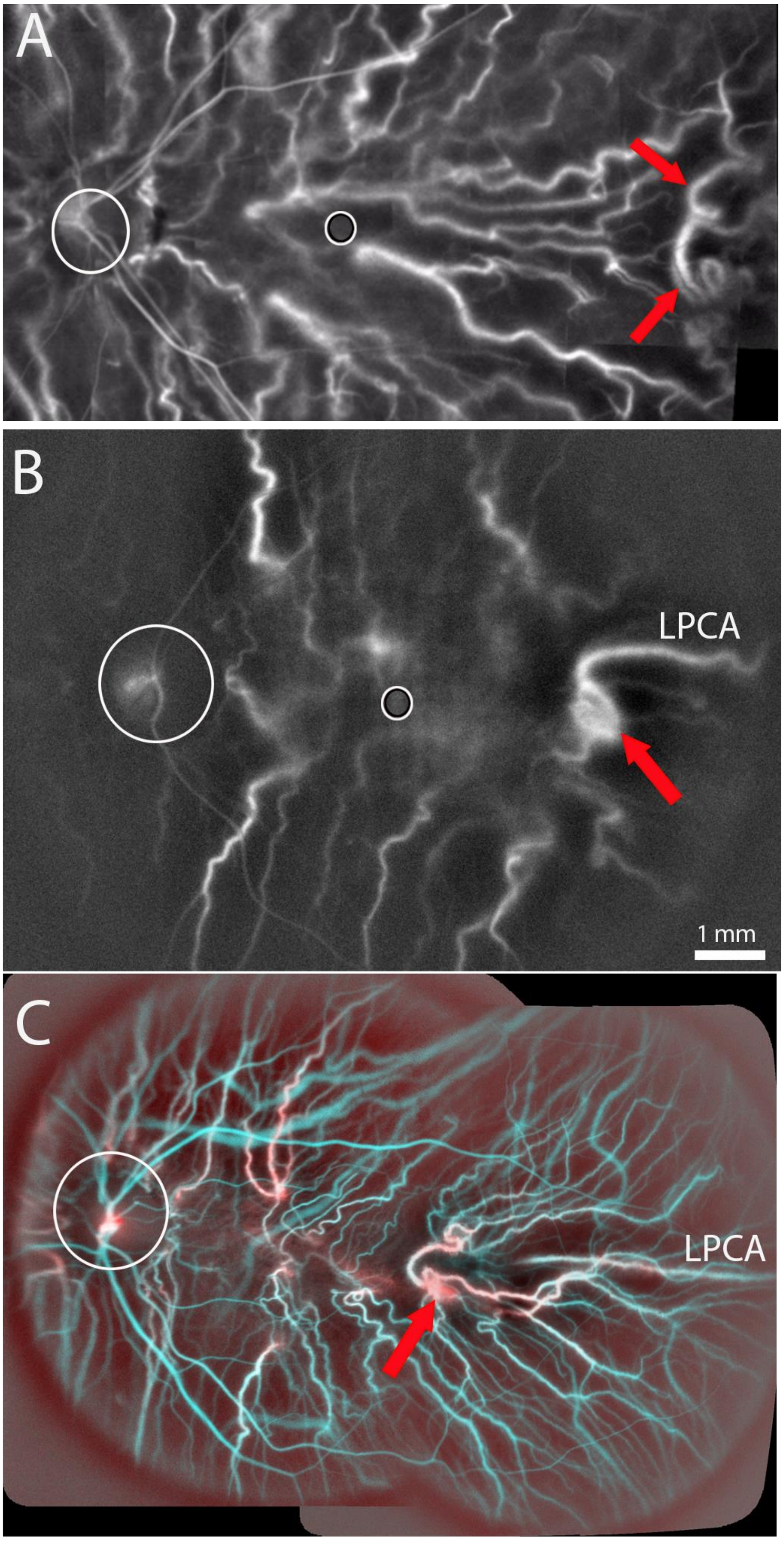

Supplementary figure 1. Example of focal arterial tortuosities (red arrows) at the émergence of LPCAs.
